# Patients with Syndrome X have normal myocardial oxygenation and perfusion compared to normal volunteers: a 3 Tesla cardiovascular magnetic resonance imaging study

**DOI:** 10.1186/1532-429X-13-S1-P126

**Published:** 2011-02-02

**Authors:** Theodoros D Karamitsos, Ranjit Arnold, Tammy J Pegg, Jane Francis, Ruairidh K Howells, Matthew D Robson, Stefan Neubauer, Michael Jerosch-Herold, Joseph B Selvanayagam

**Affiliations:** 1 OCMR Unit, University of Oxford, Oxford, UK; 2 Brigham and Women's Hospital, Boston, MA, USA; 3 Department of Cardiovascular Medicine, Flinders University, Adelaide, Australia

## Objective

The aim of our study was to assess regional myocardial perfusion and oxygenation in patients with Syndrome X and in normal volunteers, using cardiovascular magnetic resonance (CMR) imaging at 3 Tesla.

## Introduction

The pathophysiology of chest pain in patients with Syndrome X remains controversial. Previous studies using nuclear techniques or CMR to assess myocardial perfusion have shown conflicting results. Advances in perfusion imaging with CMR now enable absolute quantification of regional myocardial blood flow. Furthermore, blood oxygenation level-dependent (BOLD) CMR provides the unprecedented capability to assess regional myocardial oxygenation. We hypothesized that the combined assessment of regional perfusion and oxygenation with CMR could clarify whether patients with cardiac Syndrome X show evidence of myocardial ischemia (reduced perfusion and oxygenation) during vasodilator stress compared to normal volunteers.

## Methods

18 patients (15 women) with Syndrome X (chest pain, abnormal exercise treadmill test, normal coronary angiogram but no hypertension, diabetes or other causes of microvascular dysfunction) and 14 age and sex-matched normal volunteers (11 women) underwent CMR scanning at 3 Tesla. Myocardial function (cine CMR), scar (late gadolinium enhancement), perfusion and oxygenation were assessed. For perfusion, 2-3 short axis slices were acquired (depending on heart rate response to stress) with a saturation recovery fast-gradient echo sequence and 0.04 mmol/kg Gd-DTPA bolus. Absolute myocardial blood flow (MBF) was measured during adenosine stress (140μg/kg/min) and at rest by model independent deconvolution after saturation correction. Rest MBF was corrected for rate-pressure product. For oxygenation, using a T2-prepared blood oxygen level dependent (BOLD) SSFP sequence, signal intensity was measured at stress and rest in the slice matched to the mid-slice of the perfusion test.

## Results

All subjects successfully completed the study protocol. There were no differences in LV volumes and ejection fraction between patients and normal controls. No subject had evidence of late gadolinium enhancement. Myocardial blood flow at stress, BOLD signal change, and coronary flow reserve measurements showed no significant differences in Syndrome X patients and controls (see table 1). Oxygenation and perfusion measurements per coronary territory (LAD, LCx, RCA) were also similar between the two groups (see table).

**Table 1:** Perfusion and oxygenation measurements

	Normal (n= 14)	Syndrome X (n= 18)	*p*-value
**Global Measurements**			

Rest (corrected) MBF (ml/min/gr)	0.96±0.18	0.93±0.21	0.07
Stress MBF (ml/min/gr)	2.37±0.45	2.32±0.58	0.29
CFR	2.53±0.56	2.59±0.74	0.30
BOLD SI change (%)	17.08±7.43	17.30±7.92	0.85

**Left Anterior Descending Artery**			

Rest (corrected) MBF (ml/min/gr)	0.98±0.15	0.96 ±0.16	0.84
Stress MBF (ml/min/gr)	2.43±0.32	2.39±0.50	0.83
CFR	2.53±0.42	2.54±0.60	0.96
BOLD SI change (%)	17.4±5.90	16.7±5.61	0.74

**Left Circumflex artery**			

Rest (corrected) MBF (ml/min/gr)	0.95±0.17	0.92±0.21	0.72
Stress MBF (ml/min/gr)	2.34±0.31	2.32±0.58	0.90
CFR	2.50±0.33	2.59±0.74	0.66
BOLD SI change (%)	18.22±8.03	18.88±9.65	0.84

**Right Coronary Artery**			

Rest (corrected) MBF (ml/min/gr)	0.93±0.16	0.87±0.15	0.28
Stress MBF (ml/min/gr)	2.30±0.32	2.31±0.57	0.94
CFR	2.51±0.30	2.71±0.68	0.27
BOLD SI change (%)	16.10±7.15	17.44±6.78	0.59

BOLD, blood oxygen level-dependent; CFR, coronary flow reserve; MBF, myocardial blood flow; SI, signal intensity

## Conclusions

Patients with Syndrome X have no reduction of absolute perfusion or impairment in oxygenation during vasodilatory stress. Our findings are in contrast to other CMR studies and might reflect differences in patient population and CMR methodology (absolute vs relative quantification of perfusion).

